# A Serological Diagnosis of Coeliac Disease Is Associated with Osteoporosis in Older Australian Adults

**DOI:** 10.3390/nu10070849

**Published:** 2018-06-29

**Authors:** Michael D. E. Potter, Marjorie M. Walker, Stephen Hancock, Elizabeth Holliday, Gregory Brogan, Michael Jones, Mark McEvoy, Michael Boyle, Nicholas J. Talley, John Attia

**Affiliations:** 1 Faculty of Health and Medicine, University of Newcastle, Level 3 East, HMRI Building, Lookout Road, New Lambton Heights 2305, Australia; marjorie.walker@newcastle.edu.au (M.M.W.); Stephen.hancock@newcastle.edu.au (S.H.); liz.holliday@newcastle.edu.au (E.H.); gregory.brogan@hnehealth.nsw.gov.au (G.B.); Mark.McEvoy@newcastle.edu.au (M.M.); Michael.boyle@hnehealth.nsw.gov.au (M.B.); nicholas.talley@newcastle.edu.au (N.J.T.); john.attia@newcastle.edu.au (J.A.); 2 Australian Gastrointestinal Research Alliance (AGIRA), Newcastle 2305, Australia; 3 Department of Medicine, John Hunter Hospital, Newcastle 2305, Australia; 4 Department of Psychology, Macquarie University, Sydney 2109, Australia; mike.jones@mq.edu.au

**Keywords:** coeliac disease, osteoporosis, fractures

## Abstract

Previously thought to be mainly a disorder of childhood and early adult life, coeliac disease (CeD) is increasingly diagnosed in older adults. This may be important given the association between CeD and osteoporosis. The primary aim of this study was to determine the seroprevalence of undiagnosed CeD (‘at-risk serology’) in an older Australian community and relate this to a diagnosis of osteoporosis and fractures during a follow-up period of 12 years. We included participants from the Hunter Community Study (2004–2007) aged 55–85, who had anti-tissue transglutaminase (tTG) titres, human leukocyte antigen (HLA) genotypes, and bone mineral density measurements at baseline. Follow-up data included subsequent diagnosis of CeD and fractures using hospital information. ‘At-risk’ serology was defined as both tTG and HLA positivity. Complete results were obtained from 2122 patients. The prevalence of ‘at-risk’ serology was 5%. At baseline, 3.4% fulfilled criteria for a diagnosis of osteoporosis. During a mean of 9.7 years of follow-up, 7.4% of the cohort suffered at least one fracture and 0.7% were subsequently diagnosed with CeD. At-risk serology was significantly associated with osteoporosis in a multivariate model (odds ratio 2.83, 95% confidence interval 1.29–6.22); there was insufficient power to look at the outcome of fractures. The results of this study demonstrate that at-risk CeD serology was significantly associated with concurrent osteoporosis but not future fractures. Most individuals with a serological diagnosis of CeD were not diagnosed with CeD during the follow-up period according to medical records. Coeliac disease likely remains under-diagnosed.

## 1. Introduction

Coeliac disease (CeD), once considered rare, is now estimated to affect 1–2% of the population in Western countries [1,2]. It is an immune-mediated systemic condition, manifested by small intestinal enteropathy triggered by exposure to gluten (a complex of water insoluble proteins in wheat, rye, and barley) in the diet [1]. CeD occurs almost exclusively in those who are genetically predisposed with the haplotypes human leukocyte antigen (HLA)-DQA1*05-DQB1*02 (DQ2) and/or DQA1*03-DQB1*03:02 (DQ8) [1]. Previously thought to be a childhood disorder, CeD is increasingly diagnosed in older patients with longstanding atypical symptoms in whom the diagnosis has not previously been pursued [3,4]. The reported biopsy proven prevalence in older populations (over 55 years) has been reported to be 0.1–2.3% [5]. This may be important given the known association between CeD and diseases such as cancer and osteoporosis [6]. A recent study on case finding in the general community for individuals with undiagnosed CeD showed that these subjects were more likely to develop osteoporosis [7].

Osteoporosis is characterized by low bone mineral density (BMD) and architectural distortion of bone tissue that leads to bone fragility and an increased risk of fractures [8]. Age and gender are the major risk factors for the condition, which predominantly affect post-menopausal females [9]. Osteoporosis is a major public health problem, and over 4.7 million Australians over the age of 50 have low BMD [10]. This results in fractures, with over 140,000 fractures occurring in 2012 attributed to osteoporosis [10]. This is estimated to cost the Australian health care system over AU$3 billion per year [10]. 

Osteoporosis is common in CeD, and approximately 40% of patients with newly diagnosed CeD demonstrate a low BMD [11]. Patients with CeD are at higher risk of osteoporotic fractures [12], a risk which persists after diagnosis [13,14] for up to 20 years [15], although the absolute increase in fracture risk is low [12,16]. Importantly, BMD improves with a gluten free diet [17,18,19], although recovery is slow, taking up to 5 years to obtain complete recovery [20], in line with the slow rate of mucosal recovery in CeD [21]. The degree of adherence to the gluten free diet [18], and degree of mucosal damage at follow-up [22], has been shown to correlate with the degree of BMD recovery.

The aim of this study was to determine the seroprevalence of undiagnosed CeD in an older Australian community and relate this to a diagnosis of osteoporosis and fractures during long-term follow-up. Secondary aims included evaluation of the association between at-risk serology at baseline, and the presence of other autoimmune antibodies, and the rate of CeD diagnoses and death during the follow-up period. 

## 2. Methods

### 2.1. Ethics

The research was approved by the Human Research Ethics Committees of the Hunter New England Local Health District and the University of Newcastle, Australia.

### 2.2. Participants 

Data for this study is from the Hunter Community Study, a prospective cohort of community-dwelling older men and women (aged 55–96 years) from Newcastle, New South Wales, Australia. The sample characteristics and recruitment strategy has been described previously in detail [23]. Participants were randomly selected from the electoral roll between 2004 and 2007 and recruited using a modified Dillman strategy which included two letters of invitation followed by a phone call to non-responders. Invitation letters were sent to 9784 individuals, of whom 7575 responded and 3877 agreed to participate. A total of 3253 eventually participated in the study (a response rate of 44.5%). The sample has been shown previously to be comparable to the general Australian population in terms of gender and marital status, but is slightly younger in age [23].

### 2.3. Baseline Measures

Several self-report questionnaires were sent to participants at baseline, covering demographics, self-reported diseases, and prescribed and over the counter medication use. Anthropometric measurements including standing height (measured from the floor to the vertex of the head) and weight (measured using Tanita digital scales, Tanita, IL, USA) were taken at an initial face-to-face clinic visit. Bone mineral density was measured by heel ultrasound using the Sahara clinical bone sonometer (Holologic, Bedford, MA, USA) [24]. Osteoporosis was defined as a T score of less than or equal to −2.5 [25]. A measurement of functional capacity was performed by a ‘timed up and go test’ [26]. Physical activity was measured using a pedometer worn for seven consecutive days during waking hours to record step count [23]. Samples of serum and plasma were taken for serological measures (including anti-tissue transglutaminase (anti-tTG) antibodies, anti-nuclear antibodies (ANA), and anti-thyroid peroxidase antibodies (TPO)). Samples of serum and plasma were cryopreserved in 1 mL aliquots at −80 degrees Celsius and subsequently thawed for serological measures (including anti-tTG antibodies, anti-nuclear antibodies (ANA), and anti-thyroid peroxidase antibodies (TPO)), as well as DNA isolation and genotyping. Hazardous alcohol intake was defined for males and females, respectively, as greater than five or seven standard drinks per day or more than seven or eleven drinks on any occasion based on national guidelines [27]. Current smoking status was self-reported.

### 2.4. Follow-Up Measures

Non-traumatic fractures during follow-up were determined using linkage with hospital inpatient codes arising from contact with both public and private hospitals in the state of NSW from enrolment until 2017; data were obtained from the Centre for Health Record Linkage (CHeReL). Fractures were excluded if they were associated with a hospital code for trauma. A subsequent diagnosis of CeD was determined by self-report at follow-up contact made at 5 and 10 years and using hospital inpatient codes (ICD_10). Details regarding medications targeting low bone density (including hormone replacement therapy, selective estrogen receptor modulators, bisphosphonates, denosumab, or teriparatide) were available via linkages with the national Pharmaceutical Benefits Scheme (PBS) as well as through self-reporting at baseline and follow-up. Date of death was obtained through the National Death Index. 

### 2.5. Coeliac Serology and Genotype

Anti-tissue transglutaminase antibody levels (anti-tTG) were measured by the hospital reference laboratory on enrolment to the study, using the AESKULISA human recombinant combined Immunoglobulin A (IgA) and IgG anti-tTG assay (Aesku.Diagnostics, Wendelsheim, Germany). A cut-off of ≥25 IU/mL was considered positive in line with the local reference laboratory. HLA genotyping was performed on thawed samples using an Affymetrix Kaiser Axiom array (ThermoFisher scientific, Waltham, MA, USA). Single nucleotide polymorphisms (SNPs) on chromosome 6 were used to locate HLA-DQ-2.5 and 8. Three SNPs were selected for tagging the HLA-DQ2.2 haplotype, and haplotype phasing for the three DQ2.2 tag SNPs was performed using SHAPEIT software [28]. Those HLA-DQ2- or DQ8-positive were considered to have a permissive genotype for CeD. “At-risk serology” for CeD was defined as a combination of anti-tTG antibodies greater than or equal to 25 IU/mL, and a permissive genotype for CeD (positive HLA-DQ2.2, 2.5 or DQ8). 

### 2.6. Autoimmune Serology

ANA was assessed using HEp-2 ANA slides (Bio-Rad Laboratories, Hercules, CA, USA); ANA titre of >1/160 was defined as positive. TPO-Abs were analysed by ELISA testing (Aesku.Diagnostics, Wendelsheim, Germany).

### 2.7. Statistical Analysis

Statistical analysis was performed using STATA software (StataCorp, Texas, USA). Confidence intervals were calculated using the binomial exact method. Two models examining the association between “at-risk serology” and osteoporosis and fractures, respectively, were constructed; adjustment for several other potential risk factors was based on pre-designed directed acyclic graphs [29] (see Appendix A and Appendix B). Given that there was no difference in the mean follow-up times or mortality between at-risk serology groups, simple and multiple unconditional logistic regression was used. No multivariate analysis was conducted for the outcome of fractures according to the direct acyclic graph in order to avoid over adjustment bias for the exposure of at-risk serology (see Appendix B).

## 3. Results

### 3.1. Sample Characteristics

Of the original sample, 2121 had serum available for serology and genotype analysis and were included in the study. The included sample was slightly older (mean age 75.9 vs. 75.3 years, *p* < 0.0001) and more likely to be female than the original cohort (58.2% vs. 50.0%, *p* < 0.0001). The mean follow-up time was 9.7 years (range 0.2–12.4 years).

### 3.2. Prevalence of ‘At-Risk’ Serology, Osteoporosis, and Fracture during Follow-Up

**Figure 1:**
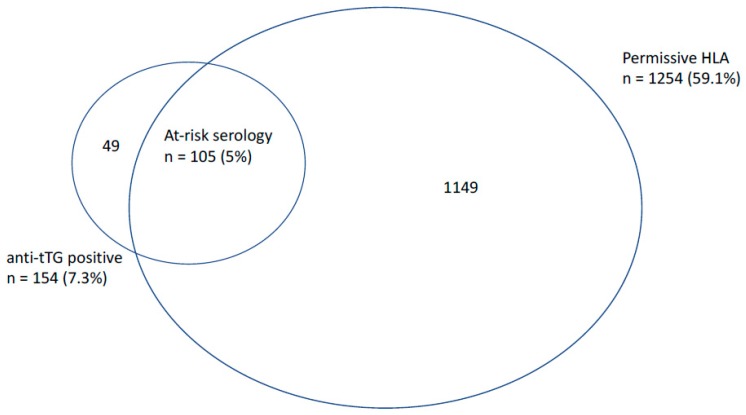
Overlap between participants with positive anti-tissue transglutaminase (anti-tTG) serology and permissive human leukocyte antigen (HLA) genotype from the overall sample of 2121 subjects.

Of the 2121 participants included in the analysis, 59.1% (95% confidence interval (CI) 56.7–61.2) had a permissive genotype and 7.3% (95%% CI 6.2–8.4) were determined to have a positive anti-tTG, with 0.8% having a high titre anti-tTG (>10 time the upper limit of normal) [21]. The mean anti-tTG was 11.4 IU/mL (range 1–313). In the entire cohort, 22.3% (95% CI 20.5–24.1) possessed at least one allele of HLA-DQ2.2, 27.2% (95% CI 25.3–29.1) possessed at least one HLA-DQ2.5 allele, and 18.9% (95% CI 17.3–20.6) possessed at least one HLA-DQ8 allele. ‘At-risk serology’ was present in 5.0% (95% CI 4.1–6.0), and 2.3% of participants who had positive anti-tTG but a non-permissive genotype for CeD (see Figure 1). Of those with a high titre anti-tTG, 88% (15/17) had a permissive HLA. There was no difference between those with and without at-risk serology in terms of mean follow-up time (*p* = 0.50).

**Figure 2:**
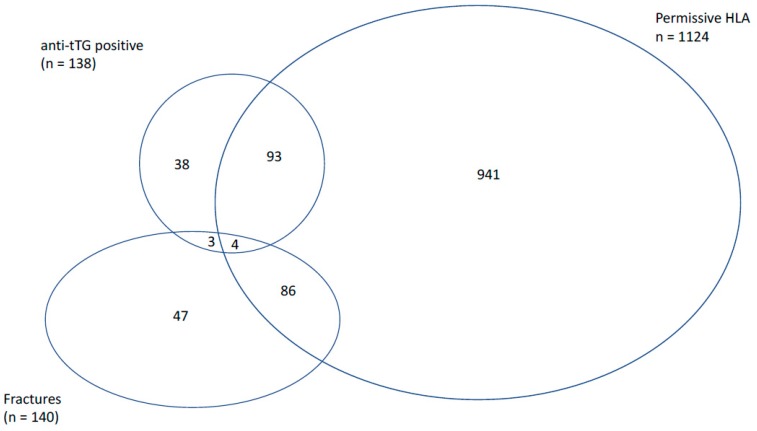
Overlap between participants with positive anti-tissue transglutaminase (tTG) serology, permissive HLA genotype, and fractures during the follow-up period from the overall sample of 1883 subjects.

A diagnosis of osteoporosis was present in 3.4% (95% CI 2.6–4.2) of participants at baseline (2.2% in males, 3.6% in females). At least one fracture (limb or other) occurred in 7.4% (95% CI 6.3–8.7) of participants (*n* = 1883) during follow-up (see Figure 2). Of those with a baseline diagnosis of osteoporosis, 10.2% received medication targeting bone density during the study period. Diagnosis of CeD during follow-up was reported in only 0.7% (95% CI 0.4–1.1) of participants (*n* = 2081), representing only 5.8% of the at-risk serology group. By the end of the follow-up period, 14.1% of the cohort had died, with no significant difference between those with and without at-risk serology (15.2% vs. 14.1%, *p* = 0.74).

### 3.3. Association between Coeliac Serology and Other Autoimmune Markers

Positive anti-tTG antibodies were associated with positive TPO antibodies but not ANA. In those with positive anti-tTG antibodies, 9.5% had a positive ANA compared with 6.8% of those without (*p* = 0.07), and 17.5% had a positive TPO antibodies compared with 10.0% without (*p* = 0.003).

### 3.4. Osteoporosis

In a univariate analysis, at-risk serology was associated with a diagnosis of osteoporosis at baseline (Odds Ratio [OR] 2.56, 95% CI 1.19–5.49) (see Table 1). Other factors significantly influencing the presence of osteoporosis at baseline included positive anti-tTG, age, gender, body mass index (BMI), and alcohol intake (see Table 1); no significant association was found for smoking or physical activity. In the multivariate model, at-risk serology, BMI, gender, smoking status, and age, but not alcohol intake, were all significantly associated with osteoporosis (see Table 2).

### 3.5. Fracture Risk 

In those with osteoporosis, 21.2% sustained a fracture during the follow-up period, compared with only 6.9% of the non-osteoporotic group (*p* < 0.001). There was no significant difference in the rate of fractures in those with at-risk serology compared to those without (4.1% vs. 7.6%, *p* = 0.2) (see Figure 2). None of the subjects with a high titre anti-tTG sustained a fracture during the follow-up period. In the univariate analysis, fracture during follow-up was also significantly associated with age and gender, but not BMI, smoking status, alcohol intake, physical activity, or the timed up and go test (see Table 3). A multivariate analysis was not performed in order to avoid over adjustment bias in accordance with the pre-constructed directed acyclic graph.

## 4. Discussion

We report a high prevalence of at-risk serology, with 5% of the sample having an elevated anti-tTG and a permissive genotype for CeD. However, 2.3% of the cohort returned a positive anti-tTG in the absence of permissive genotype, likely representing false positives. It follows that a proportion of the ‘at-risk’ group would not have CeD if gastroscopy and duodenal biopsy were undertaken. This is consistent with a previous seroprevalence study from Australia by Anderson et al. [30], which reported anti-tTG IgA antibody positivity in 5.7% of a general population cohort (median age 54–56 years in two combined cohorts), in whom approximately one-third had a non-permissive genotype for CeD. The eventual prevalence of CeD in this cohort, estimated based on biopsy or extended serological screening, was 1.2–1.9%. They suggested that in those who screen positive on anti-tTG, testing for HLA-DQ status would reduce unnecessary gastroscopies due to false positive serology by 40% [30]. Our findings of similarly high rates of positive anti-tTG in the presence of non-permissive genotype support these observations.

Second generation anti-tTG assays, employing human purified or human recombinant anti-tTG antigen (as used in our assay) have been shown to be highly sensitive and specific, with a positive predictive value of 85–100% [31,32]. These studies, however, are generally performed in cohorts with a high prevalence of disease and therefore high pre-test probabilities for an eventual diagnosis, and their performance in general population screening cohorts is likely to be lower [33]. Furthermore, there are no studies, to our knowledge, examining the performance of these assays specifically in older adults. Reports regarding false positive anti-tTG results are not uncommon, and high rates of false positivity have been demonstrated in cohorts with other autoimmune diseases [34], inflammatory bowel disease [35,36], congestive heart failure [37], and liver disease [38,39]. The link between osteoporosis and chronic diseases such as these is well established [40], and may partially explain the link between positive serology and osteoporosis, although we did not evaluate these conditions in this study. High rates of autoimmune markers have been previously reported in our own cohort, with 8% of the overall cohort testing positive for anti-TPO antibodies, and 27% testing positive for ANA antibodies [41]. We observed higher rates of both ANA and TPO antibodies in those testing positive for anti-tTG antibodies, although this result was only statistically significant for TPO antibodies and approached significance (*p* = 0.07) for ANA. There are two potential explanations for this. The first is that autoantibody findings are common in an ageing population, explaining the association between these autoantibodies and a positive anti-tTG. The second, specifically in regard to thyroid specific antibodies, is that CeD and autoimmune thyroid disease are associated [21,42], and the association between these antibodies in our cohort represents an underlying association between undiagnosed CeD and thyroid disease. Although some have called for serological diagnosis of CeD in adults (without biopsy) [43], the observations reported here do not support this diagnostic approach in older adults. 

Whilst the worldwide prevalence of CeD is thought to be 1–2% [42], this may be higher in older cohorts. A study by Vilppula et al. [6] of 2815 subjects over 55 years of age in Finland screened participants for CeD using serology, with positive cases going on to have duodenal biopsy to confirm the presence of CeD. They reported positive serology in 2.5% of the group, but only 2.1% were subsequently biopsy proven. A subset of this group was rescreened again 3 years later, of whom six had undergone seroconversion and five had developed biopsy proven CeD, representing an increase in seroprevalence to 2.7% and a biopsy proven prevalence to 2.3%, and suggesting an incidence rate of 0.08% per year [6].

The link between biopsy proven CeD and osteoporosis is well established, with a prevalence of around 40% [11]. We report an association between positive serology (in the absence of biopsy confirmation) and osteoporosis. This is consistent with previous literature which has also associated anti-tTG seropositivity with low bone mineral density. Duerksen et al. [44] retrospectively evaluated 376 women who had both coeliac serology (anti-endomysial /tTG Ab) and bone mineral density tested (with bone mineral density preceding coeliac serology by at least 6 months). They reported higher rates of osteoporosis (68% vs. 45%, *p* < 0.05) in seropositive compared with seronegative women, respectively. This has also been confirmed in a study from the USA in which undiagnosed CeD patients were more likely to develop osteoporosis and autoimmune conditions, heralding CeD in older life rather than classic malabsorption [7].

A gluten free diet improves bone mineral density in CeD patients, with complete resolution of low BMD in younger patients after two years treatment with a gluten free diet [45]. The addition of bisphosphonates, traditionally the first line pharmacologic therapy for osteoporosis, has been shown to be no more effective than a gluten free diet alone [46]. Other traditional adjunctive therapies, such as exercise, have also been shown to contribute little to BMD recovery in the context of gluten withdrawal [19]. This suggests that the pathogenesis of osteoporosis is different in CeD, and related to malabsorption of nutrients involved in bone mineralisation such as vitamin D [47], rather than hormonal regulation of bone architecture.

If indeed a relationship exists between CeD serology and osteoporosis, this may be mediated by a combination of factors, including a subset with undiagnosed CeD who develop osteoporosis secondary to enteropathy, malabsorption, and low-grade inflammation, as well as a relationship between chronic diseases that increase the likelihood of having both osteoporosis and false positive CeD serology.

There was no significant association between at-risk serology and fractures sustained during the follow-up period (20,181 person years). However, with only four participants with at-risk serology sustaining a fracture during follow-up, it is likely that the analysis was underpowered to evaluate this outcome. A systematic review by Olmos et al. [12] of case-control and cross sectional studies examining fracture risk in CeD, which included eight studies of 20,995 CeD patients and 96,777 controls, reported a fracture risk of 8.7% in CeD and 6.15% in controls (OR 1.43, 95% CI 1.15–1.78). A more recent meta-analysis by Heikkila et al. [48] limited to six prospective studies reported a slightly increased risk of bone fractures (random effects estimate: 1.30, 95% CI 1.14–1.50). Few prospective studies have examined the association between undiagnosed CeD and osteoporosis or fracture risk [48]. Agardh et al. [49] reported a study of 6480 women aged 50–64 years old (mean age 56 years) in whom both BMD (by wrist dual X-ray absorptiometry) and anti-tTG measurements were taken; they found that those with elevated tTG (≥17 IU/mL), representing approximately 0.9% of the sample, were significantly more likely to have osteoporosis (13.4% vs. 6.5%, *p* = 0.008) and fracture risk (32% vs. 19%, *p* = 0.009). 

This study has a number of strengths, including the relatively unique demographic profile of the cohort, the availability of both serological measures and bone mineral density measurements at baseline, as well as long-term follow-up allowing us to estimate fractures over a 12-year period. One significant limitation of this study is the lack of confirmatory biopsy for the diagnosis of CeD. We defined a subsequent diagnosis of CeD based on hospital coding information and self-report at follow-up as available tools of diagnosis. As mentioned, the high rate of false positivity of the anti-tTG assay means that a significant proportion of the ‘at-risk’ group are likely not to have CeD on confirmatory biopsy. Another limitation of this study is the measurement of bone mineral density, which was performed by heel ultrasound as opposed to dual X-ray absorptiometry (DXA) which is considered the gold standard [50]. Although quantitative heel ultrasound performs well against DXA in predicting fracture risk [51,52], it generally underestimates bone mineral density when compared with DXA, with lower sensitivity (21–45%) but high specificity (87–96%) at a cut-off of −2.5 for an equivalent DXA score [52]. This reflects our relatively low prevalence figures when compared to other local prevalence studies that have reported osteoporosis in 3–12% of males and 8–43% of females over age 50 [10,53].

## 5. Conclusions

At-risk coeliac serology, defined by the presence of an elevated anti-tTG antibody and a permissive genotype, is highly prevalent in older Australian adults and is significantly associated with low bone mineral density as measured by quantitative heel ultrasound. Few with at-risk serology were diagnosed with CeD during long-term follow-up. The issue of false positive serology should be addressed in studies where biopsy is used to confirm disease, as high anti-tTG levels are documented in autoimmune and liver disease. We did not find a significant relationship between at-risk serology and fractures during the follow-up period, as our analysis was underpowered for this outcome. The findings in this study would support considering the diagnosis of osteoporosis in older patients with newly diagnosed CeD.

## Figures and Tables

**Table 1:** Univariate analysis of risk factors associated with osteoporosis (OP). Risk factors are expressed as percentages in the osteoporotic and non-osteoporotic groups unless otherwise specified. CI—confidence interval. SD—standard deviation. BMI—body mass index.

	OP—%(95% CI)	No OP—%(95% CI)	Odds Ratio (95% CI)
**At-risk serology**	11.3 (3.7–18.8)	4.7 (3.8–5.7)	2.56 (1.19–5.49)
**Anti-tTG (IU/mL); mean (SD)**	20.3 (50.4)	11.1 (31.0)	1.01 (1.00–1.01)
**Positive anti-tTG**	15.5 (6.9–24.1)	7.0 (5.9–8.1)	2.44 (1.26–4.75)
**Positive HLA**	63.4 (51.9–74.9)	59.0 (56.8–61.1)	1.20 (0.74–1.97)
**Age (years); mean (SD)**	80.2 (7.4)	75.8 (7.2)	1.08 (1.05–1.11)
**BMI (kg/m^2^); mean (SD)**	27.3 (5.3)	28.8 (4.9)	0.93 (0.88–0.98)
**Gender (male)**	36.6 (25.1–48.1)	50.4 (48.3–52.6)	0.57 (0.35–0.93)
**Current smoker**	6.1 (5.1–7.2)	9.9 (2.8–17.0)	1.67 (0.75–3.72)
**Hazardous alcohol intake**	1.4 (0.0–4.2)	9.7 (8.4–10.9)	0.13 (0.02–0.97)
**Physical activity (step count per day); mean (SD)**	6160 (5106–7216)	6700 (6530–6871)	1.00 (1.00–1.00)

**Table 2:** Odds ratios from the multivariate analysis of factors associated with osteoporosis.

	Odds Ratio	95% CI	*p* Value
**At-risk serology**	3.09	1.32–7.23	0.009
**BMI**	0.94	0.89–1.00	0.04
**Gender (male)**	0.51	0.29–0.89	0.02
**Current smoking**	3.22	1.36–7.61	0.008
**Hazardous alcohol intake**	0.22	0.30–1.66	0.14
**Age**	1.08	1.04–1.12	<0.001

**Table 3:** Univariate analysis of factors associated with fractures during the follow-up period (*n* = 1, 883). Risk factors are expressed as percentages in the fracture and no-fracture groups unless otherwise specified.

	Fracture—% (95% CI)	No Fracture—% (95% CI)	Odds Ratio (95% CI)
**Osteoporosis (baseline)**	10.0 (5.0–15.0)	3.0 (2.2–3.8)	3.6 (1.9–6.7)
**At-risk serology**	2.9 (0.1–5.7)	5.3 (4.3–6.4)	0.52 (0.19–1.44)
**Anti-tTG (IU/mL); mean (SD)**	8.0 (5.1–10.8)	11.6 (10.1–13.2)	0.99 (0.98–1.00)
**Positive anti-tTG**	5.0 (1.3–8.7)	7.5 (6.3–8.8)	0.64 (0.30–1.41)
**Positive HLA**	6.4 (5.6–7.2)	5.9 (5.7–6.2)	1.2 (0.9–1.8)
**Positive tTG with non-permissive HLA**	2.3 (−0.3–4.6)	2.2 (1.5–2.9)	0.98 (0.30–3.22)
**Age (years); mean (SD)**	80.4 (79.0–81.7)	76.0 (75.6–76.3)	1.08 (1.06–1.11)
**BMI (kg/m^2^); mean (SD)**	28.9 (27.9–29.9)	28.9 (28.6–29.1)	1.00 (0.97–1.04)
**Gender (male)**	33.6 (25.7–41.5)	51.7 (49.3–54.0)	0.47 (0.33–0.68)
**Current smoker**	5.0 (1.3–8.7)	6.0 (4.9–7.1)	0.83 (0.38–1.82)
**Hazardous alcohol intake**	7.1 (2.8–11.5)	9.8 (8.4–11.2)	0.71 (0.36–1.37)
**Physical activity (step count per day); mean**	6282 (5554–7101)	6615 (6433–6797)	1.00 (1.00–1.00)
**Timed up and go test (seconds)**	10.7 (10.0–11.4)	9.9 (8.8–11.1)	1.00 (1.00–1.01)

**Figure A1:**
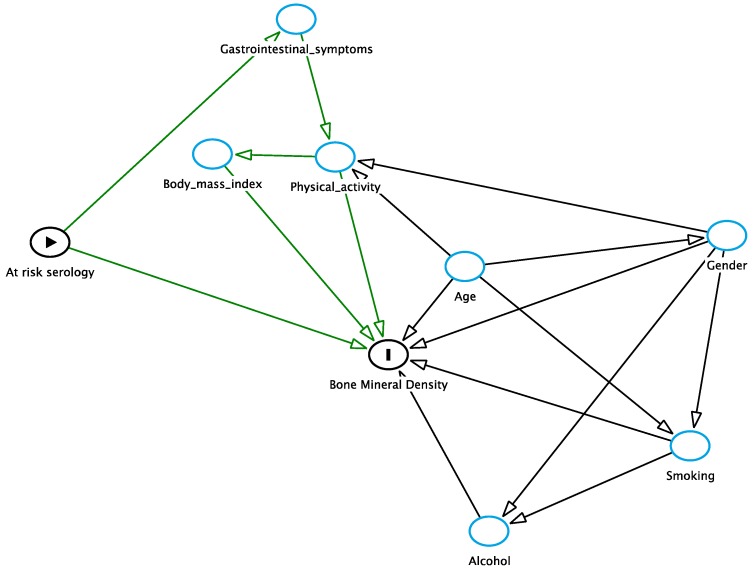
Directed acyclic graph to determine relationship between at-risk serology and osteoporosis.

**Figure A2:**
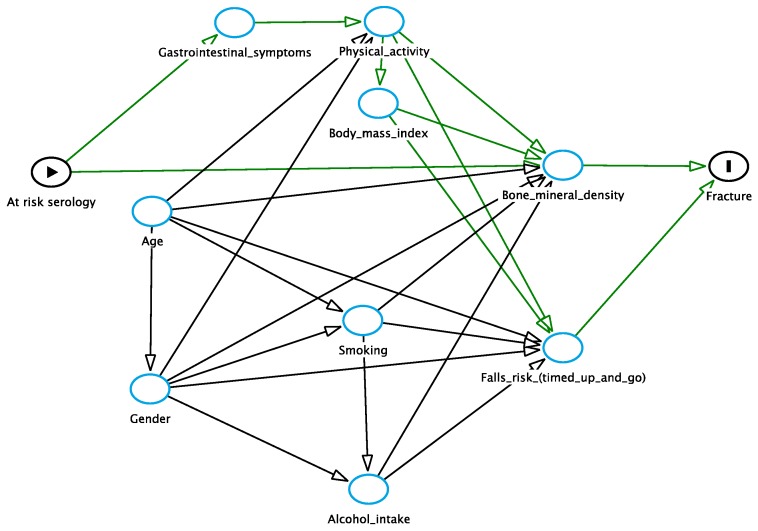
Directed acyclic graph to determine relationship between at-risk serology and fracture.
